# Perinatal and childhood outcomes of children born to female cancer survivors in South Korea

**DOI:** 10.1038/s41598-024-53088-y

**Published:** 2024-01-29

**Authors:** Ju Hyun Jin, Tae Mi Youk, Jisun Yun, Ja Yoon Heo

**Affiliations:** 1 Department of Pediatrics, National Health Insurance Service Ilsan Hospital, Goyang, Republic of Korea; 2 Research Institute, National Health Insurance Service Ilsan Hospital, Goyang, Republic of Korea; 3 Department of Obstetrics, National Health Insurance Service Ilsan Hospital, Goyang, Republic of Korea; 4 Department of Hematology-Oncology, National Health Insurance Service Ilsan Hospital, 100 Ilsan-ro, Ilsandong-gu, Goyang-si, Gyeonggi-do 10444 Republic of Korea

**Keywords:** Medical research, Oncology

## Abstract

Despite the increasing number of female cancer survivors, uncertainty remains regarding potential adverse health outcomes for their offspring. Comprehensive population-based studies would be invaluable for female cancer survivors in making decisions about their future. This study uses the National Health Information Database to investigate perinatal and long-term outcomes of offspring born to mothers with a history of cancer. In a South Korean cohort of 95,264 women aged 15–40 diagnosed with cancer between 2007 and 2010, we evaluated the outcomes of 15,221 children born to 11,092, cancer survivors. We selected 147,727 women without a history of cancer and 201,444 children as a control group. Our study found that children of female cancer survivors have a significantly higher odds ratio of primary outcomes including preterm birth, low birth weight, neonatal intensive care unit admission, and death. While there was no difference in the rate of death within 1 year of birth between the two groups, the total death rate during the follow-up period was significantly higher in children born to mothers with cancer. After adjusting for gestational age and birth weight, there was no statistically significant increased hazard ratio of secondary outcomes including cancer, chromosomal abnormalities, cerebral palsy, delayed development, epilepsy, language disorder, or hearing impairment.

## Introduction

With the advancements in cancer diagnosis and treatment, the proportion of female cancer survivors of reproductive age has increased significantly and steadily^1^. However, most of them are exposed to cancer treatment, which may have adverse effects on their reproductive health through various mechanisms^2^. Antineoplastic drugs and radiation therapy are well known to reduce ovarian function, leading to premature menopause in the worst case^3,4^. Cytotoxic drugs, such as alkylating agents and pelvic irradiation, cause DNA strand breaks and apoptosis of primordial follicles, resulting in reduced reproductive reserve^5^. Irradiation to the nervous system, such as the pituitary gland and hypothalamus, may adversely impact the neuro-endocrine axis, critical to regulating the menstrual cycle and maintaining pregnancy^6^. Chemotherapy is also known to damage the neuroendocrine axis. Cancer survivors have reported growth hormone deficiency and hypothyroidism, which can cause pubertal abnormalities and infertility^7^. Surgical removal of ovaries causes a direct reduction of ovarian reserve. Surgical removal of pelvic organs results in anatomical difficulties in conceiving and safely maintaining pregnancy^6^. Still, many female cancer survivors desire successful pregnancy and childbirth despite the potential adverse health outcomes^8^.

Even after achieving successful pregnancy, female cancer survivors may experience a significantly increased risk of preterm birth, low birth weight, and miscarriage^9,10^. Additionally, they may face uncertainties regarding their child's health, such as potentially increased risk of malformation^11^. However, the potential long-term health outcomes of children born to cancer survivors remain controversial^12,13^.

By assessing South Korean health insurance claims databases, we aimed to explore perinatal and childhood outcomes of offspring of mothers with cancer, such as cerebral palsy, delayed development, and perinatal outcomes.

## Methods

### Data source and study population

This nationwide population-based study was conducted using the National Health Information Database. All methods were performed in accordance with the relevant guidelines and regulations. The National Health Insurance Service (NHIS) is a mandatory insurance benefit system that provides healthcare to all South Korean citizens. The NHIS database contains comprehensive data on demographic characteristics, diagnosis, procedures, and costs for all inpatient and outpatient claims in South Korea. Diagnostic codes were registered by doctors according to the Korean Standard Classification of Disease (KCD), a modified version of the International Classification of Disease code. We used the 5th–7th version KCD in this study.

**Figure 1:**
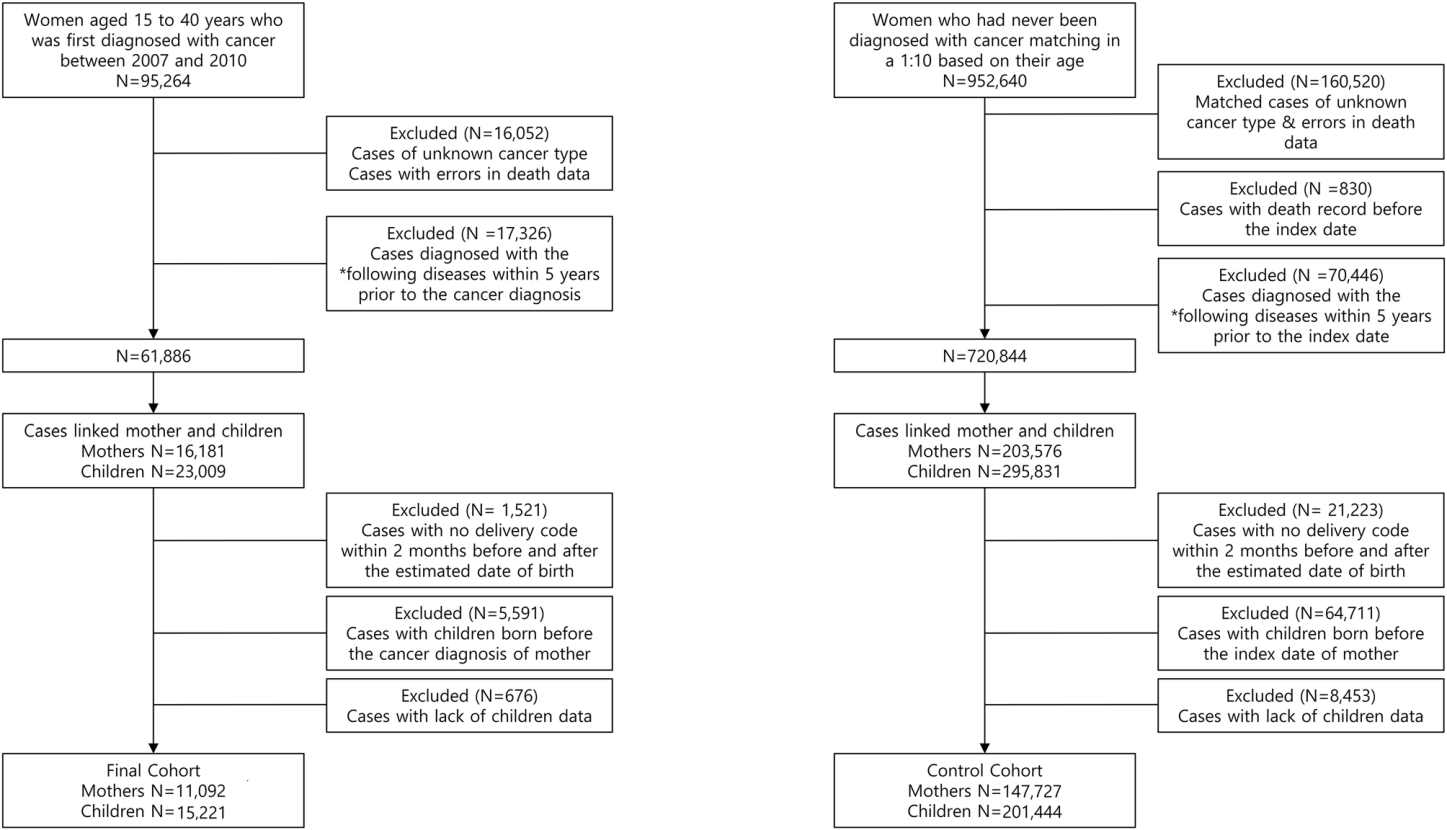
The flowchart of the study population. ^1^Benign neoplasm, borderline neoplasm, rheumatic diseases, diabetes, hypertension, chronic kidney disease, hysterectomy. ^2^Index date of the control group is the cancer diagnosis date of the matched patient in the cancer mother group.

We identified 95,264 women aged 15–40 years who were diagnosed with cancer between 2007 and 2010. We selected women who had never been diagnosed with cancer as the control group and matched them in a 1:10 ratio based on their age in 2007. Figure 1 shows the flowchart for the participants. We excluded cases with errors in data on death or cancer type and their matched controls. We also excluded cases with insufficient data and matched controls, including cases of death before the date of cancer diagnosis. Additionally, we excluded cases of congenital malformations or chromosomal anomalies, neoplasms, seropositive rheumatoid arthritis, systemic lupus erythematosus, ankylosing spondylitis, diabetes, hypertension, and chronic kidney disease and hysterectomy within 5 years prior to cancer diagnosis from both the case and control groups. Using the national health insurance card number issued to each family, we selected women with children born before January 2020 who had the same insurance number. We then excluded cases with missing or erroneous mother’s delivery code, birth before the date of cancer diagnosis (index date), or errors in the birth data. The NHIS data for children were collected from birth to December 2019. After exclusion, 11,092 women with 15,221 children were included as the case group (with cancer), and 147,727 women and 201,444 children were included as the control group (without cancer).

### Study outcomes

We assessed gestational age, birth weight, neonatal intensive care unit (NICU) admission, and death before one year as primary outcomes. Secondary outcomes included death during the follow-up period and childhood disease diagnosis in the children group. Gestational age was categorized into < 28 weeks (KCD P07.2), 28–36 weeks (KCD P07.3), and ≥ 37 weeks. Birth weight was categorized into < 1000 g (KCD P07.0), 1000–2499 g (KCD P07.1), and ≥ 2500 g. Information on NICU admission was collected from claims data. Information on whether a patient died was included in the NHIS database; however, the causes of death were not included. We identified children with one or more claims for diseases including cancer (KCD C00–C97), epilepsy (KCD G40–41), cerebral palsy (KCD G80–83), delayed development (KCD R62.0, R62.9), language disorder (KCD F80, F98.5), and chromosomal anomalies (Q90–99). Children with hearing impairment were confirmed by cochlear implantation (procedure code S5800).

### Independent variables

Demographic information of the children and their mothers, such as sex, delivery mode, residential area, income level, and maternal age, was obtained from the NHIS database. Maternal age at birth was categorized into < 25 years, 25–34 years, and ≥ 34 years. Gestational hypertension (KCD O12–15) and gestational diabetes mellitus (KCD O24) within 300 days before delivery were referred to as gestational complications. Economic levels were categorized into low-income (medical aid group and 1st–5th vigintile), lower middle-income (6th–10th vigintile), upper-middle-income (11th–15th vigintile), and high-income (16th–20th vigintile). Residential areas were divided into three: Seoul, metropolitan cities (Incheon, Busan, Daegu, Daejeon, Gwangju, and Ulsan), and others.

### Statistical analysis

Categorical data between the case and control groups were compared using the chi-square test and expressed as counts and percentages. Continuous data were compared using the t-test and expressed as means and standard deviations. The primary outcomes were binary measures, including death within the first year versus survival, gestational age less than 28 weeks versus 28–36 weeks, birth weight less than 1000 g versus 1000–2499 g, and NICU admission versus none, all obtained within one year. Therefore, a logistic regression model was used to estimate odds ratios (ORs) and 95% confidence intervals (95% CIs) to analyze the effect of maternal cancer history on children’s primary medical outcomes. Adjusted OR analysis was conducted for variables known to affect offspring’s mortality or morbidities, including sex of the child, delivery mode, maternal age at birth, economic level, and gestational complications^14^. Since the secondary outcomes are clinical events observed over a long-term period after birth, these were analyzed by hazard ratios (HRs) and 95% CIs using the Cox proportional hazard regression model. The proportional hazards assumption for the main exposure in each of our Cox models was verified using statistical tests and graphical diagnostics. Because the secondary outcomes could be attributed to birth at an earlier gestational age or lower weight at birth, we added gestational age and birth weight for further adjustment. All analyses were performed using SAS Enterprise Guide runs in SAS 9.4 (SAS Institute, Cary, NC, USA).

### Ethics approval

This study was approved by the Institutional Review Board of the National Health Insurance Service Ilsan Hospital (approval no. NHIMC-2020-01-007).

### Consent to participate/consent to publish

Institutional Review Board of the National Health Insurance Service Ilsan Hospital waived the requirement for informed consent because it uses previously collected de-identified data from National Health Insurance Service.

## Results

We identified 11,092 women aged 15–40 years, who were first diagnosed with cancer between 2007 and 2010, gave birth, and had sufficient data on their offspring (Fig. 1). We selected 201,444 women without a history of cancer and as a control group. The birth rate among women diagnosed with cancer was 11.6%, compared to 18.7% in the control group. In this cohort, thyroid cancer was the most frequent type, accounting for 36.8% **(**Table 1**)**. The mean age of the mothers at cancer diagnosis was 27.72 ± 4.21 years.

**Table 1:** Demographics of female cancer survivors who experienced childbirth.

	N	%
Total	11,092	
Cancer site		
Gastric	328	3.0
Colon	328	3.0
Lung	156	1.4
Skin	151	1.4
Breast	740	6.7
Cervical	444	4.0
Uterus	91	0.8
Ovary	1700	15.3
Kidney	72	0.6
Nervous system	234	2.1
Thyroid	4084	36.8
Hodgkin lymphoma	19	0.2
Non-Hodgkin lymphoma	155	1.4
Leukemia	79	0.7
Other	2511	22.6
Age at diagnosis		
(Mean ± SD)	27.72	4.21
Year at diagnosis		
2007	3634	32.8
2008	2611	23.5
2009	2587	23.3
2010	2260	20.4

*SD* standard deviation.

We identified 15,221 children born to a cohort of mothers with cancer (Table 2). The mean age of women at delivery in the cancer survivor group was 31.81 ± 3.90 years, and the average period since cancer diagnosis was 4.38 ± 2.68 years. The mean age of women of control group was 31.97 ± 3.91 years. The mean follow-up periods of the children in the cancer survivor and control groups were 5.86 years and 6.05 years, respectively. The cancer survivor group had significantly higher rates of cesarean sections than the control group (p = 0.0001). Mothers with cancer had a higher risk of gestational complications, including hypertension and diabetes (*p* < 0.0001). In total, 48 (0.3%) and 826 (5.4%) children were born before 28 weeks and 28–36 weeks of gestation, respectively. Children of mothers with cancer were more likely to be born preterm than children of mothers without cancer (OR 1.683 [95% CI 1.246–2.273] for less than 28 weeks and OR = 1.518 [95% CI 1.409–1.634] for 28–36 weeks of gestation). Even after adjusting for various factors, including sex of the child, delivery mode, maternal age at birth, economic level, and gestational complications, the risk of preterm birth remained. The proportion of children with low birth weight was significantly higher in children of mothers with cancer (*p* < 0.0001), with 1.3–1.6 times increased odds ratio of low birth weight after adjusting for the variables.

**Table 2:** Comparison of characteristics of children of mothers with cancer and control mothers.

	Children of mothers with cancer		Children of control mothers		*P *value
	N	%	N	%	
Total	15,221		201,444		
Male sex	7404	48.6	97,561	48.4	0.613
Gestational age					< 0.0001*
< 28 weeks	48	0.3	358	0.2	
28–36 weeks	826	5.4	7349	3.6	
≥ 37 weeks	14,347	94.3	193,710	96.2	
Birth weight					< 0.0001*
< 1000 g	50	0.3	406	0.2	
1000–2499 g	554	3.6	5488	2.7	
≥ 2500 g	14,617	96.0	195,550	97.1	
Cesarean section	6538	43.0	83,353	41.4	0.0001*
Multiple birth	759	5.0	6700	3.3	< 0.0001*
Maternal age at delivery (mean ± SD)	31.81	3.90	31.97	3.91	< 0.0001*
< 25	419	2.8	4853	2.4	< 0.0001*
25–34	11,186	73.5	145,339	72.1	
≥ 35	3616	23.8	51,252	25.4	
Maternal gestational complications	6021	39.6	74,874	37.2	< 0.0001*
Economic level					
Low	1437	9.4	20,103	10.0	< 0.0001*
Low-middle	2828	18.6	40,834	20.3	
Upper-middle	5835	38.3	80,958	40.2	
High	5121	33.6	59,549	29.6	
Residential area					
Seoul	3123	20.5	40,993	20.3	0.4978
Metropolitan cities	3753	24.7	50,528	25.1	
Others	8345	54.8	109,923	54.6	
Follow up period (mean ± SD)	5.86	2.81	6.05	2.83	< 0.0001*

*SD* standard deviation, *NICU* neonatal intensive care unit.

*p < 0.05.

Among children of mothers with cancer, the absolute risk of NICU admission was 7.13%, compared with 5.24% among children in the control group. The number of children admitted to the NICU was significantly higher among children of mothers with cancer (Table 3). The absolute risk of death within one year of birth was 0.20% for children of mothers with cancer, compared with 0.14% for children in the control group. No statistically significant increase of death was observed before 1 year of age (*p* = 0.0762). The absolute risk of total death during follow-up was 0.34% for children of mothers with cancer, compared with 0.22% for children in the control group. Death during the total follow-up period significantly increased in children born to mothers with cancer compared with those born to mothers without cancer (p = 0.0043). This association persisted after adjustment for gestational age and birth weight (adjusted OR = 1.340, 95% CI 1.002–1.793) (Table 4).

**Table 3:** Comparison of medical outcomes between children of mothers with cancer and control mothers.

	Children of mothers with cancer		Children of control mothers		*P* value
	N	%	N	%	
Total	15,221		201,444		
NICU admission	1086	7.13	10,548	5.24	< 0.001*
Death					
< 1 year	30	0.20	283	0.14	0.0762
Total	51	0.34	444	0.22	0.0043*
Cerebral palsy	89	0.58	923	0.46	0.0273*
Delayed development	482	3.17	5737	2.85	0.0232*
Epilepsy	163	1.07	2076	1.03	0.6352
Language disorder	236	1.55	3122	1.55	0.9948
Hearing impairment	7	0.05	62	0.03	0.3384
Chromosomal anomalies	23	0.15	332	0.16	0.6869
Cancer	22	0.14	304	0.15	0.8449

*NICU* neonatal intensive care unit.

*p value < 0.05.

**Table 4:** Odds ratio of medical outcomes among children of mothers with cancer and control mothers.

	Crude OR	95% CI	Adjusted OR^a^	95% CI
Gestational age				
< 28 weeks	1.683*	1.246–2.273	1.697*	1.256–2.292
28–36 weeks	1.518*	1.409–1.634	1.490*	1.383–1.605
Birth weight				
< 1000 g	1.648*	1.228–2.211	1.634*	1.217–2.194
1000–2499 g	1.351*	1.236–1.476	1.321*	1.208–1.445
NICU admission	1.390*	1.303–1.483	1.372*	1.286–1.465
Death (< 1 year)	1.404	0.963–2.046	1.405	0.963–2.048

*OR* odds ratio, *CI* confidence intervals, *NICU* neonatal intensive care unit.

^a^Adjusted for sex of child, delivery mode, maternal age at birth, economic status, and gestational complications.

*p value < 0.05.

Children of mothers with cancer were more likely to be diagnosed with cerebral palsy (HR = 1.290, 95% CI = 1.038–1.604) and delayed development (HR = 1132 [95% CI = 1.032–1.243]) (Table 5). However, the hazard ratio was not statistically significant after adjusting for gestational age and birth weight (adjusted HR = 1.122 [95% CI = 0.902–1.396] for cerebral palsy and HR = 1.053 [95% CI 0.960–1.156] for delayed development. The rates of diagnosis of epilepsy, language disorder, hearing impairment, chromosomal anomalies, and cancer were similar in children of mothers with cancer and control mothers.

**Table 5:** Hazard ratio of medical outcomes among children of female cancer survivors in comparison with children of control women.

	Crude HR	95% CI	Adjusted HR^a^	95% CI	Adjusted HR^b^	95% CI
Death (total)	1.549*	1.159–2.069	1.554*	1.163–2.077	1.340*	1.002–1.793
Cerebral palsy	1.290*	1.038–1.064	1.273*	1.024–1.582	1.122	0.902–1.396
Delayed development	1.132*	1.032–1.243	1.116*	1.017–1.225	1.053	0.960–1.156
Epilepsy	1.060	0.904–1.244	1.060	0.904–1.244	1.044	0.890–1.225
Language disorder	1.022	0.895–1.166	1.019	0.893–1.163	1.009	0.883–1.151
Hearing impairment	1.523	0.697–3.328	1.531	0.700–3.348	1.484	0.678–3.246
Cancer	0.981	0.637–1.512	0.978	0.634–1.507	0.980	0.635–1.511
Chromosomal anomalies	0.924	0.606–1.410	0.930	0.690–1.420	0.912	0.597–1.392

*HR* hazard ratio, *CI* confidence interval.

^a^Adjusted for sex of child, delivery mode, maternal age at birth, economic status, and gestational complications.

^b^Adjusted for gestational age, birth weight, sex of child, delivery mode, maternal age at birth, economic status, and gestational complications.

*p value < 0.05.

## Discussion

To our knowledge, this population-based study is the most extensive study conducted on this topic. This is the first nationwide population-based study to investigate the perinatal and childhood outcomes of children born to mothers with cancer in South Korea. We found that cancer in mothers is associated with increased the risk of preterm birth, low birth weight, NICU admission, and death in their children. The rates of cerebral palsy diagnosis and delayed development among children of mothers with cancer were higher than those among children of mothers without cancer; however, the HR was not statistically significant after adjustment for gestational age and birth weight.

The findings of this study are consistent with previous findings showing an increase in the rates of preterm birth and low birth weight in the offspring of cancer survivors^9,10,15^. There have been reports that offspring of female survivors of adolescent and young adult cancer had an increased risk of admission to a special care unit in population-based cohort studies from Western Australia^16^ and Finland^17^. Survivors are more likely to have delivery complications, such as labor dystocia, prolonged labor, fetal malpresentation, imminent fetal asphyxia, and rupture of uterus. A deteriorating uterine environment may contribute to adverse outcomes in newborns at birth, thereby increasing the need for intensive care^11,18^.

Our study showed that mortality rate in offspring born after maternal cancer diagnosis during the first year of life did not significantly increase. A previous study reported results consistent with our findings that cancer survivors did not have an elevated risk of death in their children within the first year of life^17^. However, when mortality over the entire follow-up period (5.86 ± 2.81 years in the offspring of female cancer survivors) was compared between both groups, a 1.3-fold increased risk was observed in children of cancer survivors, even after adjusting for gestational age and birth weight. A Swedish population-based study evaluated mortality risk in the offspring of women with a history of cancer at any point in their lives^19^. They reported that the overall mortality risk in the offspring of mothers diagnosed with cancer was the same as that in the background population (standardized mortality ratio [SMR] = 1.00, 95% CI 0.97–1.03). However, in this Swedish study, the relative risk of death in offspring born between 1 year before and 1 year after their mothers’ cancer diagnosis doubled during the first 4 years after birth (SMR = 2.03, 95% CI 1.46–2.68). Offspring born 1 year after maternal cancer diagnosis had significantly increased mortality risk only if the mother had hematopoietic cancer (SMR = 2.07, 95% CI 1.10–3.35). We were unable to perform further analyses because the NHIS database did not include causes of death. Other studies have reported that the main causes of death among offspring of cancer survivors are prematurity, delivery complications, and congenital anomalies^17^. Based on these results, further analyses are needed to investigate the risk factors of offspring death by dividing the subgroups according to the type of cancer, type of treatment, and timing of pregnancy.

We found that the higher risk of cerebral palsy and delayed development in children born to mothers with cancer was due to preterm birth or low birth weight. The major risk factors for cerebral palsy and delayed development are preterm birth and low birth weight^20,21^. Preventing preterm birth and low birth weight could be a fundamental intervention to improve the health outcomes of children born to cancer survivors. We also assessed the risk of epilepsy, language disorder, and hearing impairment, which are known to be increased in children born preterm^22^; however, these diseases did not show a significant increase among children of female cancer survivors. We followed the children for approximately 6 years, but a longer follow-up study would be able to evaluate other diseases whose prevalence rate increase with age.

In our study, the proportion of children with chromosomal anomalies was similar between mothers with and without cancer (0.15% vs. 0.16%). Contrary to our findings, two cohort studies conducted in Sweden reported that the risk of congenital malformation in children of female cancer survivors was significantly increased^11,23^. While our study included only chromosomal abnormalities in the analysis (Q90–99), the studies from Sweden differed by including malformations and deformities in addition to chromosomal abnormalities (Q00–99). The risk of congenital malformation among offspring was elevated in mothers diagnosed with bladder, kidney, and nervous system tumors^23,24^. However, another study in Denmark, which included only abnormal karyotypes, reported no increase in chromosomal abnormalities among children of cancer survivors^12^, which is consistent with the findings of our study.

The risk of cancer in offspring is a major concern for cancer survivors during pregnancy^25^. In a large Nordic study, the overall standardized incidence ratio (SIR) for non-retinoblastoma cancer among the offspring of cancer survivors was small but statistically significant (SIR = 1.6, 95% CI 1.1–2.4) ^26^. However, when only sporadic tumors were analyzed, the increase in SIR was not statistically significant (SIR = 1.3, 95% CI 0.8–2.0) ^26^. Another study reported that cancer risk was not high among offspring of cancer patients after the exclusion of hereditary cancer syndrome^27^. Although our study did not exclude hereditary cancers, there was no significant increase in cancer risk among offspring of cancer survivors. Given the average follow-up period of 5.86–6.05 years for children in our study, the risk of some cancers, such as lymphoma and epithelial tumors, may not have been accurately reflected. Additional research is needed on the outcomes of children born to cancer survivors with longer follow-up into adulthood.

This study has several notable strengths, including its large number of participants from a nationwide database. The NHIS is a mandatory coverage system for all citizens residing in South Korea, allowing for a thorough investigation of cancer survivors and their offspring born after cancer diagnosis. Our study was free from recall bias, as we acquired claims data of children over time rather than relying on self-reported information, providing a more in-depth understanding of long-term disease diagnosis.

We believe that our study is of interest to a broad international audience, as it provides valuable insights into the perinatal and childhood outcomes of children of female cancer survivors. However, we acknowledge that the high prevalence of thyroid cancer in the study population is a limitation that should be considered when interpreting the findings.

Despite the comprehensive nationwide coverage provided by the South Korean NHIS, the use of diagnostic codes from the NHIS claims data posed limitations in assessing disease severity. Although these codes indicate whether a disease has been diagnosed, they do not provide detailed information on the extent or severity of the condition. Additionally, incomplete or inaccurate data may result when physicians report or file claims. Regarding mothers, the NHIS database lacks important information concerning cancer, such as stage and pathology. Furthermore, the absence of detailed information on the radiotherapy location and extent of surgery precluded us from stratifying the observed findings according to maternal cancer characteristics and treatment.

Another limitation of this study is that the time of onset for some secondary outcomes, such as chromosomal anomalies or cerebral palsy, may be unclear, as we used the time of the first claim as the time of event. This is because these conditions can manifest at different times during childhood, and it may not always be possible to determine exactly when they first occurred. Despite this limitation, we believe that this study provides valuable insights into the childhood outcomes of children born to mothers with cancer.

Finally, we were unable to evaluate all potential risk factors that could influence adverse medical outcomes in this study, such as assisted reproductive technologies and pre-pregnancy chronic diseases in cancer survivors. However, we matched the study population with controls based on maternal age and adjusted for other potential confounders, such as sex, delivery method, maternal age at delivery, income, and pregnancy complications including gestational hypertension and diabetes. Additionally, we adjusted for highly correlated variables in the analysis of hazard ratios for medical outcomes among children of female cancer survivors to minimize the potential for bias.

Our study indicated that the offspring of female cancer survivors had an increased risk of preterm birth, low birth weight, NICU admission, and total death. Our findings also suggested that increased risk of cerebral palsy and delayed development may be attributed to preterm birth or low birth weight. Pregnant female cancer survivors should be closely monitored and supported with hospital access to reduce preterm birth through effective strategies, such as increased access to regular hospital checkups, immediate contact with medical staff through applications, and financial support. Healthcare professionals should also provide supportive counseling to cancer survivors who wish to conceive. When survivors prepare for pregnancy, they should be informed of the high risk of preterm birth, educated about the signs of preterm birth, and referred to centers with a NICU. Further studies are needed to identify the risk factors and underlying mechanisms that contribute to increased rates of NICU admission and mortality.

### Supplementary Information

Supplementary Information.

## Data Availability

The data that support the findings of this study are available from National Health Information Database (Republic of Korea), but restrictions apply to the availability of these data, which were used under license for the current study, and so are not publicly available. Data are however available from the corresponding author Ja Yoon Heo upon reasonable request and with permission of National Health Insurance Service (Republic of Korea).
