# Supplementary material for: Perinatal and childhood outcomes of children born to female cancer survivors in South Korea
Source: Sci Rep. 2024 Jan 29;14:2418. doi: 10.1038/s41598-024-53088-y (PMC10824740; doi:10.1038/s41598-024-53088-y)
Supplement: Supplementary file 1 — Supplementary Information. [file 41598_2024_53088_MOESM1_ESM.pdf]

Supplementary Table 1. Diagnostic codes analyzed in this study

Cancer Site	Diagnostic Codes*
Gastric	C16
Colon	C18-C20
Lung	C34
Skin	C43-C44
Breast	C50
Cervical	C53
Uterus	C54
Ovary	C56
Kidney	C64
Nervous system	C70-C72
Thyroid	C73
Hodgkin lymphoma	C81
Non-Hodgkin lymphoma	C82-C86
Leukemia	C91-C95
Other	Remaining C00-C97 diagnostic codes

*All diagnostic codes in the table were classified according to the Korean Standard Classification of Diseases (KCD).
